# Alterations of functional and structural connectivity in patients with brain metastases

**DOI:** 10.1371/journal.pone.0233833

**Published:** 2020-05-29

**Authors:** Bo Hua, Xin Ding, Minghua Xiong, Fanyu Zhang, Yi Luo, Jurong Ding, Zhongxiang Ding

**Affiliations:** 1 Artificial Intelligence Key Laboratory of Sichuan Province, Sichuan University of Science and Engineering, Zigong, China; 2 School of Automation and Information Engineering, Sichuan University of Science and Engineering, Zigong, China; 3 Department of Neurology, The First Affiliated Hospital of Chengdu Medical College, Chengdu, China; 4 Department of Radiology, Affiliated Hangzhou First People’s Hospital, Zhejiang University School of Medicine, Hangzhou, China; Hunan Normal University, CHINA

## Abstract

Metastases are the most prevalent tumors in the brain and are commonly associated with high morbidity and mortality. Previous studies have suggested that brain tumors can induce a loss of functional connectivity and alter the brain network architecture. Little is known about the effect of brain metastases on whole-brain functional and structural connectivity networks. In this study, 14 patients with brain metastases and 16 healthy controls underwent resting state functional magnetic resonance imaging (rs-fMRI) and diffusion tensor imaging (DTI). We constructed functional connectivity network using rs-fMRI signal correlations and structural connectivity network using DTI tractography. Graph theoretical analysis was employed to calculate network properties. We further evaluated the performance of brain networks after metastases resection by a simulated method. Compared to healthy controls, patients with brain metastases showed an altered “small-world” architecture in both functional and structural connectivity networks, shifting to a more randomness organization. Besides, the coupling strength of functional-structural connectivity was decreased in patients. After removing nodes infiltrated by metastases, aggravated disruptions were found in both functional and structural connectivity networks, and the alterations of network properties correlated with the removed hubs number. Our findings suggest that brain metastases interfere with the optimal network organization and relationship of functional and structural connectivity networks, and tumor resection involving hubs could cause a worse performance of brain networks. This study provides neuroimaging guidance for neurosurgical planning and postoperative assessment of brain metastases from the aspect of brain networks.

## Introduction

Metastases are the most prevalent tumors in the brain and are commonly associated with high morbidity and mortality [1]. Lung, breast, and skin are the three most common primary sites for brain metastases [2]. Possibly as a result of improvements in detection and treatment of primary cancers, the incidence of brain metastases has an increasing trend [3]. For brain metastases close to or located in eloquent areas, both itself and it’s treatment could lead to neurological deficits or cognitive dysfunctions [3,4]. The neurocognitive dysfunctions caused by tumors generally are involved in alterations of widespread functional networks rather than a focal alteration of brain functions [5].

Several neuroimaging studies have investigated the functional connectivity alteration in tumor patients. Electroencephalographic (EEG) and electrocorticographic studies reported a decrease of functional connectivity in patients with cortical lesions such as tumors [6,7].Using magnetoencephalography (MEG), Bartolomei et al. [5,8] found that brain tumors can induce diffuse, not focal, decreases in functional connectivity, and alter the network architecture of the brain. Douw et al. [9] investigated the effects of tumor treatment on functional connectivity in 15 tumor patients using MEG, and found that functional connectivity changed in a complex manner after tumor resection. Using blood oxygen level-dependent functional magnetic resonance imaging (BOLD-fMRI), previous studies have demonstrated that altered activation patterns are not only found within the lesion regions but are often present in distant brain regions in tumor patients [10,11]. Thus, functional connectivity analysis can help us to understand the effect of a brain tumor on spatially distributed but temporally correlated network in the brain [12].

In addition to functional connectivity, structural connectivity in tumor patients could also be altered when brain tumors are close to or located in the white matter of the brain. Therefore, it is crucial to know the alterations of functional and structural connectivity networks in tumor patients. Functional connectivity network can be constructed by using EEG, MEG or BOLD-fMRI [13]. Unlike EEG and MEG based functional connectivity networks which were measured by extra-cerebral sensors, functional connectivity network by using BOLD-fMRI depicts temporal dependencies between distinct brain regions [14]. Structural connectivity network based on white matter tracts quantified by diffusion tractography [15], gives insights into microstructural white matter architecture. Prior studies have proved that structural connectivity is highly predictive of and places constraints on functional interactions across the human brain network [16,17]. Accordingly, structural connectivity is considered as the physical substrate of functional connectivity [17]. In turn, functional connectivity may influence structural connectivity through brain plasticity [18]. The relationship (coupling) between functional and structural connectivity has been found to enhance with age during normal development [18], and disrupt in disease states [19–21]. So, it could be more sensitive to detect subtle brain pathophysiological abnormalities by using the coupling of functional-structural connectivity than any single modality [21]. Moreover, the human brain network is an efficient complex network with a “small-world” topology [22]. Using graph analysis, Bartolomei et al. [5] found that tumor patients showed a changed “small-world” network architecture in the functional connectivity network. Assessment at network level allows to inspect topological properties of normal and pathological brain networks [23].

On the basis of the aforementioned findings, we predict that brain metastases may alter network topological characteristics as well as the relationship of functional and structural connectivity. Therefore, one aim of the present work is to evaluate the impact of brain metastases on whole-brain functional and structural connectivity networks. For patients with less than three metastases, surgical resection is generally considered as a valid treatment to extend the survival time [3]. However, little is known about whether regions infiltrated by metastases can be resected since its resection may induce neurocognitive impairments [24]. Thus, our second aim is to investigate changes of functional and structural connectivity networks after tumor removal and further assess the association between these changes and the removed region numbers from the aspect of brain networks.

## Materials and methods

### Participants

This study included 16 patients (13 males, mean age: 61.00 ± 7.80 years) with newly diagnosed brain metastatic tumors and 16 healthy controls (11 males, mean age: 57.13 ± 10.92 years), matched in age (*p* = 0.1861, two-tailed Mann-Whitney U test) and sex (*p* = 0.6851, two-tailed Fisher’s exact test). The recruitment was performed from September 2016 to May 2017. Some of these participants have been used in our previous study [25]. For each subject, we performed a conventional MRI protocol for routine investigation, including T1/T2-weighted imaging, T2-weighted fluid-attenuated inversion recovery (FLAIR) imaging and diffusion weighted imaging. The patients were recruited from the department of neurosurgery at Zhejiang Provincial People's Hospital, Hangzhou Medical College. The inclusion criteria were: 1) the age was greater than or equal to 18 years; 2) the number of metastases was no more than three; 3) the patients had known primary tumor sites; 4) the patients had no history of brain surgery or other neurological disease (e.g. traumatic brain injury, stroke or other focal brain lesions); 5) the patients had no intra-tumoral hemorrhage or prior cerebral hemorrhage; 6) the patients had no significant peritumoral brain edema. Then, one patient with five metastases and one with obvious encephalomalacia caused by prior cerebral hemorrhage were excluded, and 14 brain metastatic patients were therefore used for the following analyses. Out of 14 patients, nine patients had suffered from a single brain metastasis and five from two to three brain metastases. Thirteen patients had the primary tumor site in the lung and one in the rectum. Demographic and clinical data of brain metastases are shown in S1 Table. The healthy controls were recruited from the staff of Zhejiang Provincial People's Hospital, Hangzhou Medical College. The inclusion criteria included: 1) no gross brain abnormalities in brain MRI images; 2) no history of neurological or psychiatric disorders.

The study was approved by the Medical Ethics Committee of Zhejiang Provincial People's Hospital, Hangzhou Medical College, Hangzhou, China. The methods used in this study were performed in accordance with the Declaration of Helsinki. Written informed consent was obtained from each participant prior to enrollment in the experiments. Authors had no access to information that could identify individual participants during or after data collection.

### Image acquisition

All imaging data were collected on a 3.0-T MR scanner (Discovery 750; GE Healthcare, Milwaukee, Wis). During data acquisition, subjects were instructed to relax with their eyes closed, not to fall asleep, and to keep their heads still. Foam padding and earplugs were used to minimize head motion and scanner noise. The resting state fMRI (rs-fMRI) data were acquired by using an echo-planar-imaging sequence with the following parameters: repetition time [TR]/echo time [TE] = 2000/30 ms, field of view = 220×220 mm^2^, matrix = 64×64, voxel size = 3.44×3.44×3.2 mm^3^, 35 axial slices without slice gap, flip angle = 90°, and a total of 210 volumes for each subject. The diffusion tensor imaging (DTI) data were collected by using a single-shot echo-planar-imaging sequence, including 25 volumes with diffusion sensitizing gradients applied along 25 non-collinear directions (b = 1000 s/mm^2^) and one volume without diffusion weighting (b = 0 s/mm^2^). The acquisition parameters were: TR/TE = 8637/64.1 ms, field of view = 96×96 mm^2^, matrix = 128×128, voxel size = 0.75×0.75×1.5 mm^3^, 81 axial slices without slice gap, and flip angle = 90°. The high-resolution T1-weighted structural images were also acquired by using a magnetization-prepared rapid gradient-echo sequence with the following parameters: TR/ TE = 6.652/2.928 ms, field of view = 256×256 mm^2^, matrix = 256×256, voxel size = 1×1×1mm^3^, 192 sagittal slices without slice gap, and flip angle = 12°. Here, the acquisition parameters for the rs-fMRI and T1-weighted structural data were the same as in our previous study [25].

### Brain network construction

#### Network node definition

We employed the automated anatomical labeling (AAL) template [26] to parcellate the whole brain into 90 cortical and subcortical regions (S2 Table). Furthermore, we constructed a high-resolution network with 1024 regions. See S1 Text for details. The two parcellation schemes were defined as AAL-90 and AAL-1024 respectively and applied to the following network analyses, as our previous study [20].

#### Edge definition of functional connectivity network

Functional images were corrected for temporal differences and head motion, and then normalized to MNI space. For the patients, we additionally used a cost-function modification to avoid transformation bias since the tumor tissue may lead to distortions during normalization [27,28]. Then, several nuisance parameters were regressed out of the data at each brain voxel. The resulting residuals were temporally band-pass-filtered (0.01–0.1 Hz). We acquired the correlation matrix for each subject using Pearson correlation analysis. The absolute correlation coefficient |*r*_*ij*_| between region *i* and *j* was defined as the weighted edge *w*_*ij*_ of functional connectivity network. Since the mechanisms of negative functional connectivity are still less understood [29], we also constructed weighted functional connectivity network with only positive correlation coefficient and performed the following network analyses. See S1 Text for details.

#### Edge definition of structural connectivity network

Diffusion weighted images were corrected for head motions and eddy current distortions. The diffusion tensor models were then estimated by using the Diffusion Toolkit [30], and fiber tracking was implemented in the DTI native space using Fiber Assignment by Continuous Tracking (FACT) algorithm [31]. For structural connectivity network, nodes were defined in the DTI native space [20,32]. Here, a cost-function modification was also additionally used to avoid tumor-induced transformation bias in the patients [27,28]. We constructed weighted structural connectivity network and defined its weighted edge *w*(*e*) as: $w\left(e\right)=2/\left(S_{i}+S_{j}\right)\sum_{f\in F_{e}}1/l\left(f\right)$. *S*_*i*_ and *S*_*j*_ are two-dimension intersects of the individual’s white matter with AAL region *i* and *j*, respectively [20,21]; *F*(*e*) refers to the fibres set connecting regions *i* and *j*; *l*(*f*) refers to the length of the fiber *f*. Structural connectivity weights were further scaled by the maximum of this matrix in each subject [33]. See S1 Text for details.

### Network topological analysis

We performed graph theoretical analysis to compute network topological properties via the Brain Connectivity Toolbox (http://www.brain-connectivtiy-toolbox.net) [34]. The global network properties such as connectivity strength *S*_*net*_, normalized weighted clustering coefficient *γ*, normalized weighted characteristic path length *λ* and the small-worldness *σ* were evaluated. To determine the global role of each node in the brain networks, we also computed the nodal properties including nodal connectivity strength *S*_*i*_, efficiency *E*_*i*_ and normalized betweenness centrality *b*_*i*_. Nodes with high connectivity strength, efficiency or betweenness centrality (> mean + SD) were considered as global hubs in the brain network [35]. See S1 Text for details.

### Coupling analysis of functional-structural connectivity

In line with our previous studies [20,21], we constrained the coupling analysis by the edges with existing (non-zero) structural connectivity. The coupling of functional-structural connectivity was obtained by calculating Pearson’s correlation between functional and structural connectivity values. See S1 Text for details.

### Network analysis after tumors removal

To describe the influence of tumor resection on brain functional and structural networks, a simulated procedure was performed. Firstly, we identified the nodes infiltrated by brain tumors in each patient. Then, we computed the functional-structural connectivity coupling, and global network properties of functional and structural networks after removing these nodes and the corresponding connections for each patient.

### Statistical analysis

The network topological parameters may change with the threshold selection. To comprehensively evaluate the tumor-induced network changes, we computed network topological properties using a range of cost thresholds (0.1≤cost≤0.26 for the AAL-90 scheme, 0.019≤cost≤0.036 for the AAL-1024 scheme) (S1 Text). To avoid possible bias on network analysis from single threshold, we further computed the area under the curve (AUC) of network topological properties.

For group comparisons of global network properties and the coupling of functional-structural connectivity between controls and patients, two-sample two-tailed *t*-test was performed. The statistical significance for these group comparisons was determined using a nonparametric permutation test method [33,36]. The permutations were performed 5000 times to test whether the group differences were significant. A threshold of *α* = 0.05 was used for testing network properties and coupling strength.

Furthermore, paired-samples *t*-test was employed to assess the difference of global network properties and the functional-structural connectivity coupling in patients before and after tumor removal. The significance threshold strategy was carried out as above. To further evaluate the relationship of altered network properties and coupling strength with the tumor removal, Spearman correlation analysis was performed between these network alterations and the number of hubs infiltrated by tumors.

## Results

### Hub distributions

**Fig 1:**
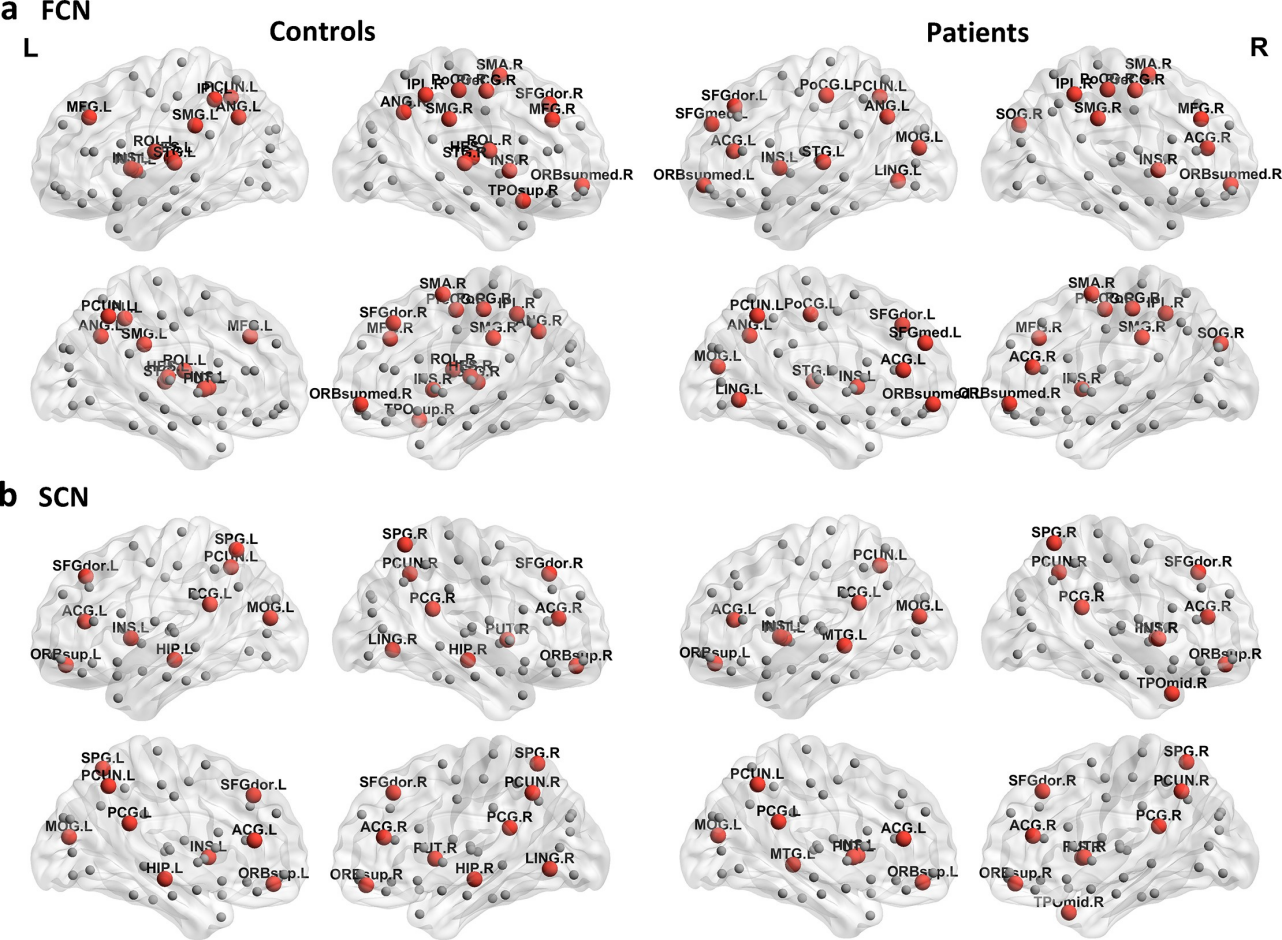
Hub distributions of AAL-90 FCN and SCN in healthy controls and patients with brain metastases. The results are visualized with the BrainNet viewer (NKLCNL, Beijing Normal University, China). (a) functional connectivity network, twelve hubs were identified in both healthy controls and patients, including bilateral insula, left precuneus, angular gyrus, superior temporal gyrus, right precentral gyrus, middle frontal gyrus, supplementary motor area, medial orbital part of superior frontal gyrus, postcentral gyrus, inferior parietal gyrus and supramarginal gyrus. (b) structural connectivity network, thirteen hubs were identified in both healthy controls and patients, including bilateral orbital part of superior frontal gyrus, anterior cingulate gyrus, posterior cingulate gyrus, precuneus, left insula, middle occipital gyrus, right dorsolateral part of superior frontal gyrus, superior parietal gyrus and putamen. The red spheres and the grey dots denote hub and non-hub regions, respectively. The nodal regions are located according to their centroid stereotaxic coordinates. FCN, functional connectivity network; SCN, structural connectivity network; L, left; R, right.

We calculated nodal properties and identified global hubs which had high connectivity strength, efficiency or betweenness centrality. The hub distributions were similar to those reported in previous studies [15,20,32,35,37]. For the functional connectivity network under AAL-90 parcellation, twelve nodes were identified as global hubs in both controls and patients, including bilateral insula, left precuneus, angular gyrus, superior temporal gyrus, right precentral gyrus, middle frontal gyrus, supplementary motor area, medial orbital part of superior frontal gyrus, postcentral gyrus, inferior parietal gyrus and supramarginal gyrus (Fig 1A). For the structural connectivity network under AAL-90 parcellation, thirteen nodes were considered as hubs in both controls and patients, including bilateral orbital part of superior frontal gyrus, anterior cingulate gyrus, posterior cingulate gyrus, precuneus, left insula, middle occipital gyrus, right dorsolateral part of superior frontal gyrus, superior parietal gyrus and putamen (Fig 1B). The similar hub distributions were found in the functional and structural connectivity networks using the AAL-1024 scheme (S1 Fig). In addition, we also displayed the hub distributions of functional connectivity networks constructed by only positive correlation coefficients in the two groups (S2 Fig), which were partly different to those of functional connectivity networks constructed by absolute correlation coefficients.

### Global topological properties of brain networks

**Fig 2:**
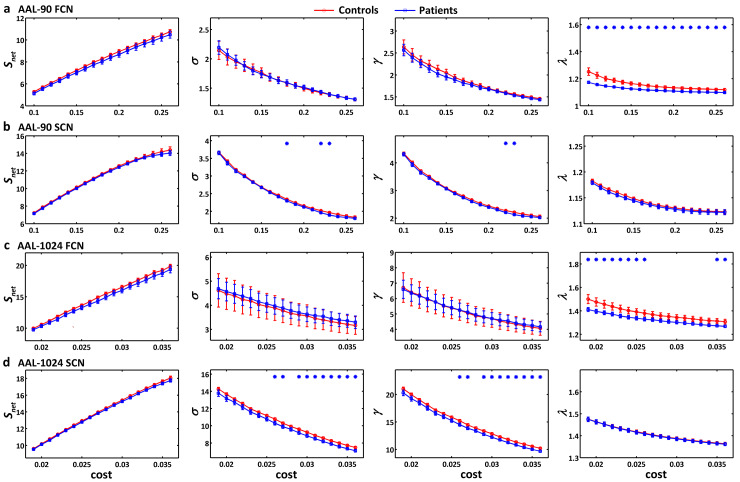
Global network properties of brain networks as a function of cost threshold. (a) functional connectivity network under AAL-90 scheme. (b) structural connectivity network under AAL-90 scheme. (c) functional connectivity network under AAL-1024 scheme. (d) structural connectivity network under AAL-1024 scheme. The vertical bar indicates the standard deviation across subjects. The asterisks indicate the statistically significant difference between healthy controls and patients (permutation testing, *p*<0.05). FCN, functional connectivity network; SCN, structural connectivity network.

**Fig 3:**
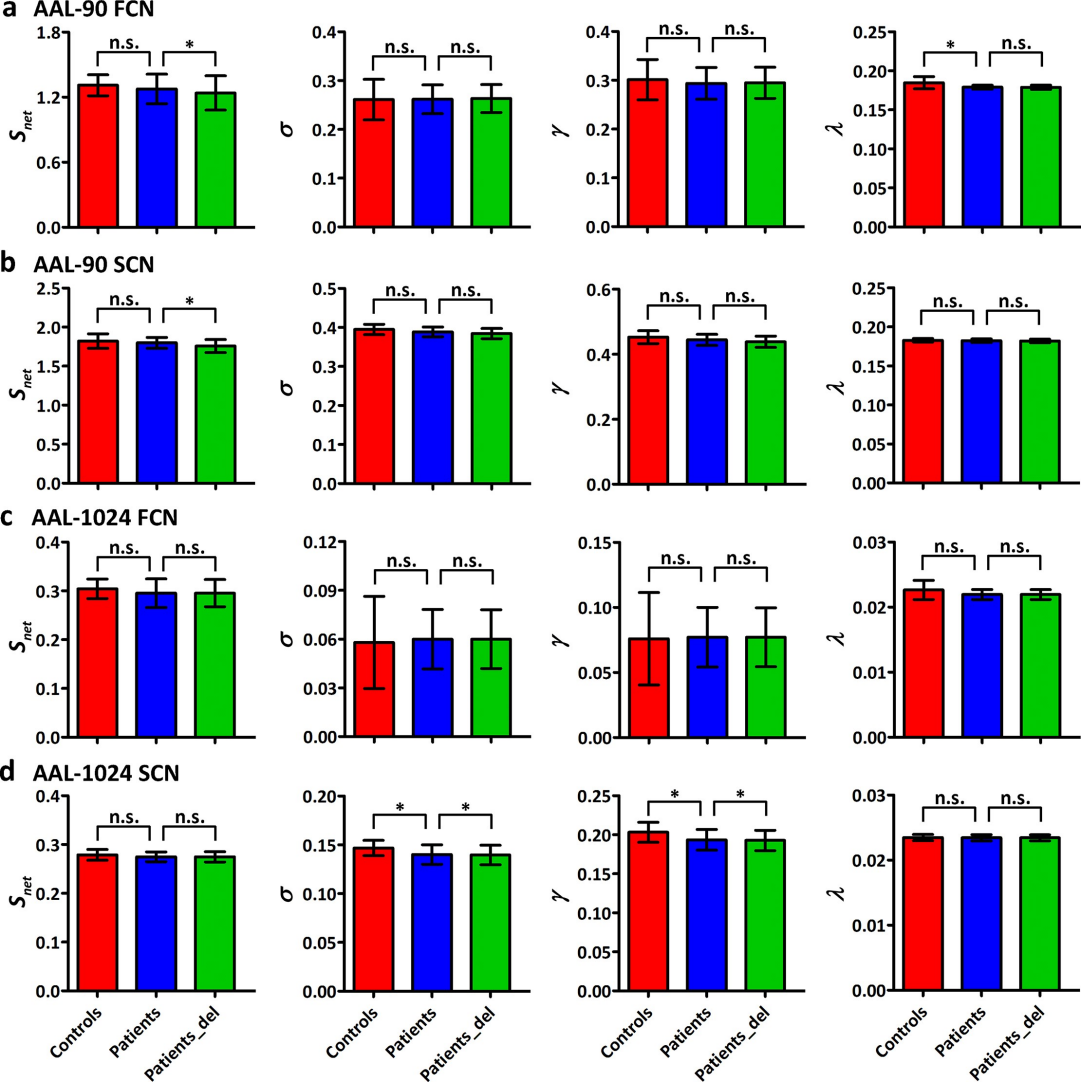
Comparison results of integrated AUC for each global network property. (a) functional connectivity network under AAL-90 scheme. (b) structural connectivity network under AAL-90 scheme. (c) functional connectivity network under AAL-1024 scheme. (d) structural connectivity network under AAL-1024 scheme. Two-sample two-tailed *t*-test was performed for group comparison between healthy controls and patients. Paired-samples *t*-test was employed for group comparison in patients before and after tumor removal. The vertical bar indicates the standard deviation across subjects. The asterisks indicate the statistically significant group difference (permutation testing, *p*<0.05). FCN, functional connectivity network; SCN, structural connectivity network; n.s., no significant difference; patients_del, patients after tumor deletion.

For both controls and patients, a small-world organization (*σ*>1) was found in functional and structural connectivity networks under either AAL-90 parcellation or AAL-1024 parcellation (Fig 2). Additionally, functional connectivity networks constructed by both AAL-90 and AAL-1024 schemes in patients showed a decreased normalized characteristic path length *λ*, while structural connectivity network constructed by AAL-1024 scheme had a decreased small-worldness *σ* and normalized clustering coefficient *γ* (permutation testing, *p*<0.05) (Fig 2). To compare the integrated AUC of each global network property, we found a similar patterns of topological alterations in patients: decreased normalized characteristic path length *λ* for the functional connectivity network under AAL-90 parcellation, and decreased small-worldness *σ* and normalized clustering coefficient *γ* for the structural connectivity network under AAL-1024 parcellation (permutation testing, *p*<0.05) (Fig 3).

**Fig 4:**
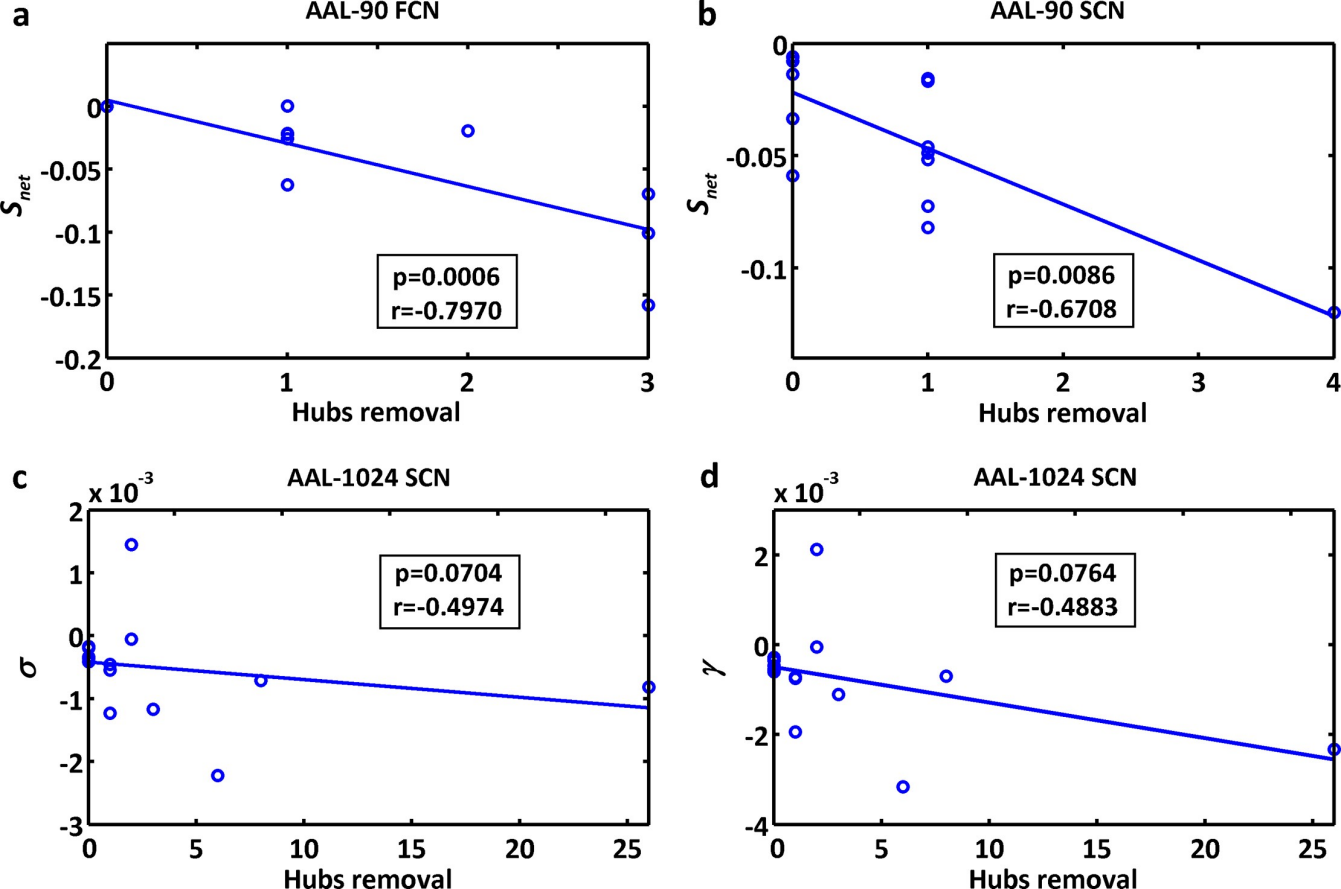
Correlations between altered network properties and the removed hubs number in patients with brain metastases. (a) altered network connectivity strength *S*_net_ of AAL-90 FCN negatively correlated with the removed hubs number (*r* = −0.7970, *p* = 0.0006). (b) altered network connectivity strength *S*_net_ of AAL-90 SCN negatively correlated with the removed hubs number (*r* = −0.6708, *p* = 0.0086). (c) altered small-worldness *σ* of AAL-1024 SCN showed a negative correlation trend with the removed hubs number (*r* = −0.4974, *p* = 0.0704). (d) altered normalized clustering coefficient *γ* of AAL-1024 SCN showed a negative correlation trend with the removed hubs number (*r* = −0.4883, *p* = 0.0764). FCN, functional connectivity network; SCN, structural connectivity network.

After removing the nodes infiltrated by tumors in patients, some significant alterations were found: decreased connectivity strength *S*_net_ for both functional and structural connectivity networks under AAL-90 parcellation, and decreased small-worldness *σ* and normalized clustering coefficient *γ* for the structural connectivity network under AAL-1024 parcellation (permutation testing, *p*<0.05) (Fig 3). The alteration of connectivity strength *S*_net_ was negatively correlated with the number of removed hubs for both functional and structural connectivity networks under AAL-90 parcellation (*r* = −0.7970, *p* = 0.0006 for the former; *r* = −0.6708, *p* = 0.0086 for the later). There was a negative correlation trend between the removed hubs number and the alteration of the small-worldness *σ* (*r* = −0.4974, *p* = 0.0704) and normalized clustering coefficient *γ* (*r* = −0.4883, *p* = 0.0764) for the structural connectivity network under AAL-1024 parcellation (Fig 4).

We also computed and compared the global topological properties of functional connectivity networks constructed by only positive correlation coefficients in the two groups. Consistent with the results from the functional connectivity network constructed by absolute correlation coefficients, a decreased normalized characteristic path length *λ* under AAL-90 parcellation was found in patients compared to controls (S3 and S4 Figs). After removing the nodes infiltrated by tumors in patients, we also found a decreased connectivity strength *S*_net_ under AAL-90 parcellation (S4 Fig), which negatively correlated with the number of removed hubs (*r* = −0.8546, *p*<0.001) (S5 Fig).

### Functional-structural connectivity coupling

**Fig 5:**
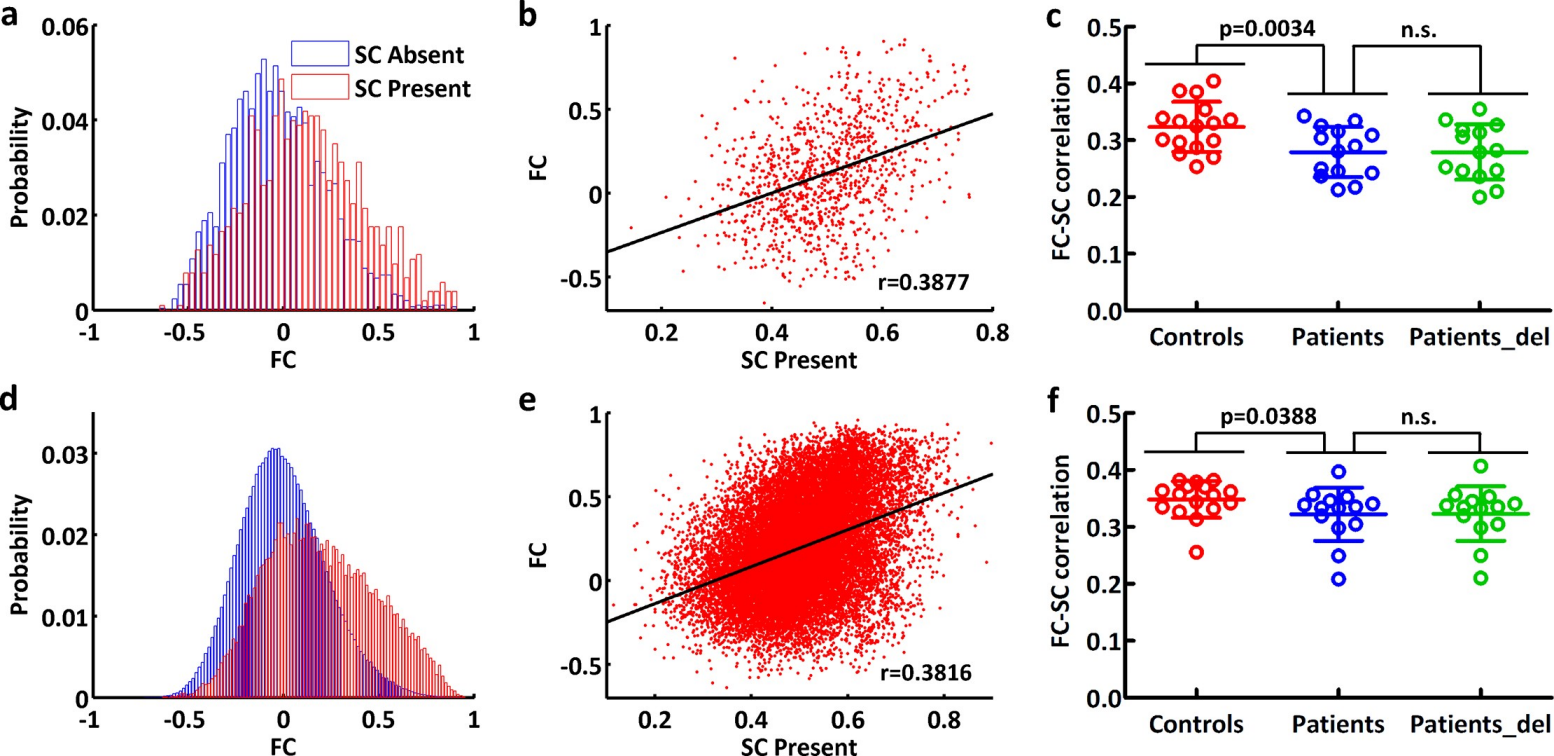
Functional-structural connectivity coupling under AAL-90 scheme (a-c) and AAL-1024 scheme (d-f). (a, d) the probability densities of functional connectivity values between structurally connected and unconnected region pairs for a selected participant. (b, e) scatter plot of functional connectivity against non-zero structural connectivity for a selected participant. (c, f) coupling strength in healthy controls, patients and patients after tumor removal. Two-sample two-tailed *t*-test was performed for group comparison between healthy controls and patients. Paired-samples *t*-test was employed for group comparison in patients before and after tumor removal. FC, functional connectivity; SC, structural connectivity; n.s., no significant difference; patients_del, patients after tumor deletion.

Functional connection values distributed over a wide range whether there existed structural connections or not. Constrained by existing structural connections, a positive correlation was found between functional connectivity values and structural connectivity values in each subject. These features of functional-structural connectivity coupling were found for both AAL-90 and AAL-1024 parcellations (Fig 5).

Compared to controls (0.3236±0.0440 for AAL-90 and 0.3482±0.0322 for AAL-1024), the patients with brain metastases (0.2790±0.0444 for AAL-90 and 0.3224±0.0467 for AAL-1024) revealed a significant decrease in the coupling strength of functional-structural connectivity (*p* = 0.0034 for AAL-90; *p* = 0.0388 for AAL-1024). There was no significant difference of the coupling strength in patients before and after tumor removal for neither AAL-90 nor AAL-1024 parcellations (Fig 5C and 5F).

## Discussion

This is the first study to investigate the effect of brain metastases on both functional and structural connectivity networks using graph theoretical analysis. Our main findings are as follows: (i) patients with brain metastases had an altered “small-world” architecture in both functional and structural connectivity networks, suggesting a more randomness organization of brain networks; (ii) the coupling strength between functional and structural connectivity networks was significantly decreased in patients; (iii) after removing nodes infiltrated by tumors, some network properties showed a more decrease in patients, and the alteration negatively correlated with the removed hubs number. These findings demonstrate a tumor-induced alteration of network organization and relationship between functional and structural connectivity, providing neuroimaging guidance for neurosurgical planning and postoperative assessment of brain metastases from the aspect of brain networks.

In the present study, both functional and structural connectivity networks exhibited a small-world topology in either healthy controls or patients with brain metastases. Small-world topology reflects a general organization principle in the human brain networks, allowing global and local parallel information processing [22,38]. This topology has been thought to sustain our normal cognitive functioning [39,40].

Nevertheless, some altered topological patterns were found in patients with brain metastases compared to healthy controls: a decrease of small-worldness *σ* and normalized clustering coefficient *γ* for the structural connectivity network. The small-world network represents an optimal organization between regular and random networks [41]. Thus, our results indicate that structural connectivity network in patients with brain metastases has a less optimal topological organization (lower *σ*), and shifts to a more random network architecture (lower *γ*). In a previous study based on MEG, Bartomomei et al. [5] found that functional connectivity network showed a tendency to more random configuration in brain tumor patients. Similarly, our result of reduced normalized characteristic path length *λ* for the functional connectivity network also suggest a more random organization in patients with brain metastases. Unlike structural connectivity network, no differences were found for the small-worldness *σ* and normalized clustering coefficient *γ* in functional connectivity network between controls and patients. Structural connectivity of the adult mammalian brain can essentially keep constant for a long time without brain lesion, while functional connectivity can substantially reconfigure within a very short time [16]. Brain metastases may lead to shift or even damage of anatomical regions or white matter tracts nearby [42], and then directly alter the network topology of structural connectivity. However, functional connectivity could be rapidly reconfigured to response to the influence of tumor. Moreover, the reduced normalized characteristic path length *λ* could favor the speed of global information processing [22]. Therefore, the topological alterations of functional connectivity network (a significant decrease for *λ*, but no difference for *σ* and *γ*) may reflect a compensatory mechanism to the less optimal organization of structural connectivity network in patients with brain metastases.

Combining rs-fMRI and DTI techniques, we investigated the relationship of functional and structural connectivity networks in patients with brain metastases and healthy controls. It is proved that structural connectivity is highly predictive of and places constraints on functional interactions, while functional connectivity may influence structural connectivity through brain plasticity [18]. The relationship between functional and structural connectivity is complex, since strong functional connectivity still exists without direct structural connectivity [43]. Thus, similar to our previous studies [20,21], we only investigated the function-structure relations on the direct (non-zero) structural connections. Previous studies have reported a reconfigured relationship of functional and structural connectivity under either physiological [16,18], or pathological states [19–21]. Consistently, we found a decreased coupling strength of functional-structural connectivity in patients with brain metastases. The finding indicates a loose relationship of functional and structural connectivity, and may reflect an abnormal mechanism of brain networks induced by brain metastases.

To assess the influence of tumor resection on functional and structural connectivity networks, a simulated procedure was performed to evaluate the network performance after removing the nodes infiltrated by brain metastases. We found a further decrease of network connectivity strength *S*_net_ for both functional and structural connectivity networks, as well as a further decrease of the small-worldness *σ* and normalized clustering coefficient *γ* for the structural connectivity network in patients after metastases removal. *S*_net_ measures the global connectivity strength in the brain networks [15]. *σ* and *γ* are important indices to evaluate whether the network is optimal for global and local parallel information processing [22,38]. Thus, our results reflect a worse network performance in patients after metastases removal, which may be attributed to the removal of some important regions like “hubs” infiltrated by metastases. Hubs are thought to be crucial for the global coordination of information flow and maintaining network integrity [44]. Previous studies have demonstrated a more deleterious effect of hub lesions on information flow and network stability in both normal and diseased state [35,44,45]. Indeed, we found a negative correlation of these altered topological properties with the number of removed hubs in patients. Our findings indicate that the more hubs are removed during metastases resection, the worse performance brain networks have, which should be paid special attention to when designing surgical plan.

Previous studies have reported negative functional connectivity in the resting state [46,47], but the mechanisms of negative functional connectivity are still less understood [29]. To avoid uncertainty, we also computed and compared network properties of weighted functional connectivity network constructed by only positive correlation coefficients in the two groups. Interestingly, although the hubs distributions were partly different, the results of altered network properties in patients before and after metastases resection were consistent with those for the functional connectivity network constructed by absolute correlation coefficients. These findings may suggest that the topological organization of functional connectivity network is intrinsic whether considering negative functional connectivity or not.

It is worth mentioning that the decreased connectivity strength *S*_net_ for both functional and structural connectivity networks were found only under AAL-90 parcellation not under AAL-1024 parcellation after metastases resection, which could be related to the number of removed hubs in patients. The percent of removed hubs under AAL-90 parcellation (≤3.3% for the functional connectivity network, ≤4.4% for the structural connectivity network) was larger than that under AAL-1024 parcellation (≤0.7% for the functional connectivity network, ≤2.5% for the structural connectivity network) in patients. According to the definition of hubs in this study, the hubs generally have large connectivity strength. Thus, the decreased connectivity strength was found under AAL-90 parcellation due to the relatively large percent of removed hubs, which could be further verified by the negative correlation of the decreased connectivity strength with the removed hubs number. Besides, the decreased small-worldness *σ* and normalized clustering coefficient *γ* for the structural connectivity network were found only using the AAL-1024 scheme not using the AAL-90 scheme in patients before and after metastases removal. The “small-world” parameters (e.g. small-worldness and clustering coefficient) have been demonstrated to vary largely in value under different parcellation scales [48]. Therefore, our findings indicate that finer parcellation scale could be more sensitive to capture alternations of network organization, and network analyses on different parcellation scales can provide more comprehensive information in disease state.

There are some limitations in the current study. First, the sample size of patients was relatively small. Even so, some statistically significant alterations of brain networks were detected. It should be noted that these preliminary findings need to be verified in a large sample size in future study. Second, there was a low overlap of metastases locations in our relatively small sample size. Subgroup analyses based on tumor locations in a large sample size may provide more specific guidance for neurosurgical planning and postoperative assessment of brain metastases. Third, there exists biased sex proportion (thirteen males vs. one female) in patients with brain metastases. Previous studies have reported the effect of sex differences on functional and structural connectivity networks [37,49–51]. Although no statistically significant difference was found in sex between controls and patients, there should be some reservation about the extent to which our findings can be generalized to patients with brain metastases as a whole. Finally, we assessed the influence of tumor resection on brain networks by a simulated tumor (node) removal. However, it is difficult to know the real changes of brain networks, especially functional connectivity networks, resulting from tumor resection only by a simple node removal method. Recently, Wang et al. [52] proposed a critical dynamical model method and successfully simulated functional connectivity matrices from structural connectivity matrices, which may provide a new idea to simulate changes of functional connectivity networks after node removal. Overall, the current results need to be verified by using postoperative neuroimaging data in future work.

## Conclusions

This study for the first time assessed the effect of brain metastases on both functional and structural connectivity networks using graph theoretical analysis. Patients with brain metastases showed an altered topological organization as well as a decreased coupling strength of functional and structural connectivity networks. Moreover, the removal of metastases aggravated disruptions of functional and structural connectivity networks in patients, and the alterations of network properties correlated with the removed hubs number. Overall, our results indicate that brain metastases interfere with the optimal network organization and relationship of functional and structural connectivity networks, and the hubs removal could lead to a worse performance of brain networks.

## Supporting information

S1 Checklist STROBE statement—checklist of items that should be included in reports of observational studies. (DOCX)

S1 Text Supplement methods. (PDF)

S1 Table Demographic and clinical characteristics of brain metastases. (PDF)

S2 Table Regions of interest (ROI) in the AAL template. (PDF)

S1 Fig Hub distributions of AAL-1024 FCN and SCN in healthy controls and patients with brain metastases. (a) functional connectivity network. (b) structural connectivity network. The results are visualized with the BrainNet viewer (NKLCNL, Beijing Normal University, China). The results of AAL-1024 scheme show similar pattern with those of AAL-90 scheme. The red spheres and the grey dots denote hub and non-hub regions, respectively. The nodal regions are located according to their centroid stereotaxic coordinates. FCN, functional connectivity network; SCN, structural connectivity network; L, left; R, right.
(TIF)

S2 Fig Hub distributions of FCN constructed by only positive correlation coefficients in the two groups. (a) under AAL-90 scheme. (b) under AAL-1024 scheme. The results are visualized with the BrainNet viewer (NKLCNL, Beijing Normal University, China). The hub distributions of AAL-1024 scheme is similar with those of AAL-90 scheme. The red spheres and the grey dots denote hub and non-hub regions, respectively. The nodal regions are located according to their centroid stereotaxic coordinates. FCNpos, functional connectivity network constructed by only positive correlation coefficients; L, left; R, right.
(TIF)

S3 Fig Global network properties of FCN constructed by only positive correlation coefficients under different cost thresholds. (a) under AAL-90 scheme. (b) under AAL-1024 scheme. The vertical bar indicates the standard deviation across subjects. The asterisks indicate the statistically significant difference between healthy controls and patients (permutation testing, *p*<0.05) FCNpos, functional connectivity network constructed by only positive correlation coefficients.
(TIF)

S4 Fig Integrated AUC for each global network property in FCN constructed by only positive correlation coefficients. (a) under AAL-90 scheme. (b) under AAL-1024 scheme. Two-sample two-tailed *t*-test was performed for group comparison between healthy controls and patients. Paired-samples *t*-test was employed for group comparison in patients before and after tumor removal. The vertical bar indicates the standard deviation across subjects. The asterisks indicate the statistically significant group difference (permutation testing, *p*<0.05). FCNpos, functional connectivity network constructed by only positive correlation coefficients; n.s., no significant difference; patients_del, patients after tumor deletion.
(TIF)

S5 Fig Correlations between altered connectivity strength and the removed hubs number in patients with brain metastases. The altered network connectivity strength *S*_net_ of AAL-90 FCN constructed by only positive correlation coefficients negatively correlated with the removed hubs number (*r* = −0.8546, *p*<0.0001). FCNpos, functional connectivity network constructed by only positive correlation coefficients.
(TIF)
