# Supplementary material for: Alterations of functional and structural connectivity in patients with brain metastases
Source: PLoS One. 2020 May 29;15(5):e0233833. doi: 10.1371/journal.pone.0233833 (PMC7259727; doi:10.1371/journal.pone.0233833)
Supplement: S1 Checklist — (DOCX) [file pone.0233833.s001.docx]

STROBE Statement—checklist of items that should be included in reports of observational studies

|  | Item No. | Recommendation | Page  No. | Relevant text from manuscript |
| --- | --- | --- | --- | --- |
| **Title and abstract** | 1 | (*a*) Indicate the study’s design with a commonly used term in the title or the abstract | p2 | In this study, 14 patients with brain metastases and 16 healthy controls underwent resting state functional magnetic resonance imaging (rs-fMRI) and diffusion tensor imaging (DTI). |
|  |  | (*b*) Provide in the abstract an informative and balanced summary of what was done and what was found | p2 | We constructed functional connectivity network using rs-fMRI signal correlations and structural connectivity network using DTI tractography.-- We further evaluated the performance of brain networks after metastases resection by a simulated method.-- Compared to healthy controls, patients with brain metastases showed an altered “small-world” architecture in both functional and structural connectivity networks. Besides, the coupling strength of functional-structural connectivity was decreased in patients. After removing nodes infiltrated by metastases, aggravated disruptions were found in both functional and structural connectivity networks, and the alterations of network properties correlated with the removed hubs number. |
| Introduction | | | |  |
| Background/rationale | 2 | Explain the scientific background and rationale for the investigation being reported | p3-4 | The neurocognitive dysfunctions caused by tumors generally are involved in alterations of widespread functional networks rather than a focal alteration of brain functions. Several neuroimaging studies have investigated the functional connectivity alteration in tumor patients. It could be more sensitive to detect subtle brain pathophysiological abnormalities by using the coupling of functional-structural connectivity than any single modality |
| Objectives | 3 | State specific objectives, including any prespecified hypotheses | p4-5 | One aim of the present work is to evaluate the impact of brain metastases on whole-brain functional and structural connectivity networks. Our second aim is to investigate changes of functional and structural connectivity networks after tumor removal. |
| Methods | | | |  |
| Study design | 4 | Present key elements of study design early in the paper | p5 | This study included 16 patients (13 males, mean age: 61.00 ± 7.80 years) with newly diagnosed brain metastatic tumors and 16 healthy controls (11 males, mean age: 57.13 ± 10.92 years), matched in age ($p=0.1861$, two-tailed Mann-Whitney U test) and sex ($p=0.6851$, two-tailed Fisher’s exact test). |
| Setting | 5 | Describe the setting, locations, and relevant dates, including periods of recruitment, exposure, follow-up, and data collection | p5 | The recruitment was performed from September 2016 to May 2017. The patients were recruited from the department of neurosurgery at Zhejiang Provincial People's Hospital. The healthy controls were recruited from the staff of Zhejiang Provincial People's Hospital. |
| Participants | 6 | (*a*) *Cohort study*—Give the eligibility criteria, and the sources and methods of selection of participants. Describe methods of follow-up  *Case-control study*—Give the eligibility criteria, and the sources and methods of case ascertainment and control selection. Give the rationale for the choice of cases and controls  *Cross-sectional study*—Give the eligibility criteria, and the sources and methods of selection of participants | p5 | This study included 16 patients (13 males, mean age: 61.00 ± 7.80 years) with newly diagnosed brain metastatic tumors and 16 healthy controls (11 males, mean age: 57.13 ± 10.92 years), matched in age ($p=0.1861$, two-tailed Mann-Whitney U test) and sex ($p=0.6851$, two-tailed Fisher’s exact test). The patients were recruited from the department of neurosurgery at Zhejiang Provincial People's Hospital. The inclusion criteria were: 1) the age was greater than or equal to 18 years; 2) the number of metastases was no more than three; 3) the patients had known primary tumor sites; 4) the patients had no history of brain surgery or other neurological disease (e.g. traumatic brain injury, stroke or other focal brain lesions); 5) the patients had no intra-tumoral hemorrhage or prior cerebral hemorrhage; 6) the patients had no significant peritumoral brain edema. The healthy controls were recruited from the staff of Zhejiang Provincial People's Hospital. The inclusion criteria included: 1) no gross brain abnormalities in brain MRI images; 2) no history of neurological or psychiatric disorders. |
|  |  | (*b*) *Cohort study*—For matched studies, give matching criteria and number of exposed and unexposed  *Case-control study*—For matched studies, give matching criteria and the number of controls per case | p5 | This study included 16 patients (13 males, mean age: 61.00 ± 7.80 years) with newly diagnosed brain metastatic tumors and 16 healthy controls (11 males, mean age: 57.13 ± 10.92 years), matched in age ($p=0.1861$, two-tailed Mann-Whitney U test) and sex ($p=0.6851$, two-tailed Fisher’s exact test). |
| Variables | 7 | Clearly define all outcomes, exposures, predictors, potential confounders, and effect modifiers. Give diagnostic criteria, if applicable | p5 | For each subject, we performed a conventional MRI protocol for routine investigation, including T1/T2-weighted imaging, T2-weighted fluid-attenuated inversion recovery (FLAIR) imaging and diffusion weighted imaging. The patients were recruited from the department of neurosurgery at Zhejiang Provincial People's Hospital. The inclusion criteria were: 1) the age was greater than or equal to 18 years; 2) the number of metastases was no more than three; 3) the patients had known primary tumor sites; 4) the patients had no history of brain surgery or other neurological disease (e.g. traumatic brain injury, stroke or other focal brain lesions); 5) the patients had no intra-tumoral hemorrhage or prior cerebral hemorrhage; 6) the patients had no significant peritumoral brain edema. |
| Data sources/ measurement | 8* | For each variable of interest, give sources of data and details of methods of assessment (measurement). Describe comparability of assessment methods if there is more than one group | p5 | This study included 16 patients (13 males, mean age: 61.00 ± 7.80 years) with newly diagnosed brain metastatic tumors and 16 healthy controls (11 males, mean age: 57.13 ± 10.92 years), matched in age ($p=0.1861$, two-tailed Mann-Whitney U test) and sex ($p=0.6851$, two-tailed Fisher’s exact test). The patients were recruited from the department of neurosurgery at Zhejiang Provincial People's Hospital. The inclusion criteria were: 1) the age was greater than or equal to 18 years; 2) the number of metastases was no more than three; 3) the patients had known primary tumor sites; 4) the patients had no history of brain surgery or other neurological disease (e.g. traumatic brain injury, stroke or other focal brain lesions); 5) the patients had no intra-tumoral hemorrhage or prior cerebral hemorrhage; 6) the patients had no significant peritumoral brain edema. The healthy controls were recruited from the staff of Zhejiang Provincial People's Hospital. The inclusion criteria included: 1) no gross brain abnormalities in brain MRI images; 2) no history of neurological or psychiatric disorders. |
| Bias | 9 | Describe any efforts to address potential sources of bias | p5-7 | This study included 16 patients (13 males, mean age: 61.00 ± 7.80 years) with newly diagnosed brain metastatic tumors and 16 healthy controls (11 males, mean age: 57.13 ± 10.92 years), matched in age ($p=0.1861$, two-tailed Mann-Whitney U test) and sex ($p=0.6851$, two-tailed Fisher’s exact test). Functional images were corrected for temporal differences and head motion, and then normalized to MNI space. For the patients, we additionally used a cost-function modification to avoid transformation bias since the tumor tissue may lead to distortions during normalization. Diffusion weighted images were corrected for head motions and eddy current distortions. |
| Study size | 10 | Explain how the study size was arrived at | p5 | This study included 16 patients (13 males, mean age: 61.00 ± 7.80 years) with newly diagnosed brain metastatic tumors and 16 healthy controls (11 males, mean age: 57.13 ± 10.92 years), matched in age ($p=0.1861$, two-tailed Mann-Whitney U test) and sex ($p=0.6851$, two-tailed Fisher’s exact test). The recruitment was performed from September 2016 to May 2017. The patients were recruited from the department of neurosurgery at Zhejiang Provincial People's Hospital. Then, one patient with five metastases and one with obvious encephalomalacia caused by prior cerebral hemorrhage were excluded, and 14 brain metastatic patients were therefore used for the following analyses. The healthy controls were recruited from the staff of Zhejiang Provincial People's Hospital. |

Continued on next page

| Quantitative variables | 11 | Explain how quantitative variables were handled in the analyses. If applicable, describe which groupings were chosen and why | p8-9 | The coupling of functional-structural connectivity was obtained by calculating Pearson’s correlation between functional and structural connectivity values. Then, we computed the functional-structural connectivity coupling, and global network properties of functional and structural networks after removing these nodes and the corresponding connections for each patient. To comprehensively evaluate the tumor-induced network changes, we computed network topological properties using a range of cost thresholds (0.1≤cost≤0.26 for the AAL-90 scheme, 0.019≤cost≤0.036 for the AAL-1024 scheme) (S1 Text). To avoid possible bias on network analysis from single threshold, we further computed the area under the curve (AUC) of network topological properties. |
| --- | --- | --- | --- | --- |
| Statistical methods | 12 | (*a*) Describe all statistical methods, including those used to control for confounding | p9 | To comprehensively evaluate the tumor-induced network changes, we computed network topological properties using a range of cost thresholds. To avoid possible bias on network analysis from single threshold, we further computed the area under the curve (AUC) of network topological properties. For group comparisons of global network properties and the coupling of functional-structural connectivity between controls and patients, two-sample two-tailed *t*-test was performed. The statistical significance for these group comparisons was determined using a nonparametric permutation test method. Furthermore, paired-samples *t*-test was employed to assess the difference of global network properties and the functional-structural connectivity coupling in patients before and after tumor removal. Spearman correlation analysis was performed between these network alterations and the number of hubs infiltrated by tumors. |
|  |  | (*b*) Describe any methods used to examine subgroups and interactions | NA |  |
|  |  | (*c*) Explain how missing data were addressed | NA |  |
|  |  | (*d*) *Cohort study*—If applicable, explain how loss to follow-up was addressed  *Case-control study*—If applicable, explain how matching of cases and controls was addressed  *Cross-sectional study*—If applicable, describe analytical methods taking account of sampling strategy | p5 | This study included 16 patients (13 males, mean age: 61.00 ± 7.80 years) with newly diagnosed brain metastatic tumors and 16 healthy controls (11 males, mean age: 57.13 ± 10.92 years), matched in age ($p=0.1861$, two-tailed Mann-Whitney U test) and sex ($p=0.6851$, two-tailed Fisher’s exact test). |
|  |  | (*e*) Describe any sensitivity analyses | NA |  |
| Results | | | | |
| Participants | 13* | (a) Report numbers of individuals at each stage of study—eg numbers potentially eligible, examined for eligibility, confirmed eligible, included in the study, completing follow-up, and analysed | p5 | This study included 16 patients (13 males, mean age: 61.00 ± 7.80 years) with newly diagnosed brain metastatic tumors and 16 healthy controls (11 males, mean age: 57.13 ± 10.92 years). Then, one patient with five metastases and one with obvious encephalomalacia caused by prior cerebral hemorrhage were excluded, and 14 brain metastatic patients were therefore used for the following analyses. |
|  |  | (b) Give reasons for non-participation at each stage | p5 | Then, one patient with five metastases and one with obvious encephalomalacia caused by prior cerebral hemorrhage were excluded, and 14 brain metastatic patients were therefore used for the following analyses. |
|  |  | (c) Consider use of a flow diagram | NA |  |
| Descriptive data | 14* | (a) Give characteristics of study participants (eg demographic, clinical, social) and information on exposures and potential confounders | p5 | Demographic and clinical data of brain metastases are shown in S1 Table. |
|  |  | (b) Indicate number of participants with missing data for each variable of interest | NA |  |
|  |  | (c) *Cohort study*—Summarise follow-up time (eg, average and total amount) | NA |  |
| Outcome data | 15* | *Cohort study*—Report numbers of outcome events or summary measures over time | NA |  |
|  |  | *Case-control study—*Report numbers in each exposure category, or summary measures of exposure | p5 | This study included 16 patients (13 males, mean age: 61.00 ± 7.80 years) with newly diagnosed brain metastatic tumors and 16 healthy controls (11 males, mean age: 57.13 ± 10.92 years). Then, one patient with five metastases and one with obvious encephalomalacia caused by prior cerebral hemorrhage were excluded, and 14 brain metastatic patients were therefore used for the following analyses. S1 Table |
|  |  | *Cross-sectional study—*Report numbers of outcome events or summary measures | NA |  |
| Main results | 16 | (*a*) Give unadjusted estimates and, if applicable, confounder-adjusted estimates and their precision (eg, 95% confidence interval). Make clear which confounders were adjusted for and why they were included | p11-13 | functional connectivity networks constructed by both AAL-90 and AAL-1024 schemes in patients showed a decreased normalized characteristic path length $\lambda$, while structural connectivity network constructed by AAL-1024 scheme had a decreased small-worldness $\sigma$ and normalized clustering coefficient $\gamma$ (permutation testing, $p<0.05$). After removing the nodes infiltrated by tumors in patients, some significant alterations were found: decreased connectivity strength $S_{\text{net}}$ for both functional and structural connectivity networks under AAL-90 parcellation, and decreased small-worldness $\sigma$ and normalized clustering coefficient $\gamma$ for the structural connectivity network under AAL-1024 parcellation (permutation testing,$p<0.05$). The alteration of connectivity strength $S_{\text{net}}$ was negatively correlated with the number of removed hubs for both functional and structural connectivity networks under AAL-90 parcellation. Compared to controls ($0.3236\pm0.0440$for AAL-90 and $0.3482\pm0.0322$ for AAL-1024), the patients with brain metastases ($0.2790\pm0.0444$ for AAL-90 and $0.3224\pm0.0467$ for AAL-1024) revealed a significant decrease in the coupling strength of functional-structural connectivity ($p=0.0034$ for AAL-90; $p=0.0388$ for AAL-1024). |
|  |  | (*b*) Report category boundaries when continuous variables were categorized | NA |  |
|  |  | (*c*) If relevant, consider translating estimates of relative risk into absolute risk for a meaningful time period | NA |  |

Continued on next page

| Other analyses | 17 | Report other analyses done—eg analyses of subgroups and interactions, and sensitivity analyses | NA |  |
| --- | --- | --- | --- | --- |
| Discussion | | | | |
| Key results | 18 | Summarise key results with reference to study objectives | p14 | Our main findings are as follows: (i) patients with brain metastases had an altered “small-world” architecture in both functional and structural connectivity networks, suggesting a more randomness organization of brain networks; (ii) the coupling strength between functional and structural connectivity networks was significantly decreased in patients; (iii) after removing nodes infiltrated by tumors, some network properties showed a more decrease in patients, and the alteration negatively correlated with the removed hubs number. |
| Limitations | 19 | Discuss limitations of the study, taking into account sources of potential bias or imprecision. Discuss both direction and magnitude of any potential bias | p16-17 | There are some limitations in the current study. First, there was a low overlap of metastases locations in our relatively small sample size. Subgroup analyses based on tumor locations in a large sample size may provide more specific guidance for neurosurgical planning and postoperative assessment of brain metastases. Second, there exists biased sex proportion (thirteen males vs. one female) in patients with brain metastases. Although no statistically significant difference was found in sex between controls and patients, there should be some reservation about the extent to which our findings can be generalized to patients with brain metastases as a whole. Finally, we assessed the influence of tumor resection on brain networks by a simulated tumor (node) removal. However, it is difficult to know the real changes of brain networks resulting from tumor resection only by a simulation method. The current results need to be verified by using postoperative neuroimaging data in future work. |
| Interpretation | 20 | Give a cautious overall interpretation of results considering objectives, limitations, multiplicity of analyses, results from similar studies, and other relevant evidence | p14-17 | These findings demonstrate a tumor-induced alteration of network organization and relationship between functional and structural connectivity. Our findings indicate that the more hubs are removed during metastases resection, the worse performance brain networks have, which should be paid special attention to when designing surgical plan. Although no statistically significant difference was found in sex between controls and patients, there should be some reservation about the extent to which our findings can be generalized to patients with brain metastases as a whole. |
| Generalisability | 21 | Discuss the generalisability (external validity) of the study results | p17 | our results indicate that brain metastases interfere with the optimal network organization and relationship of functional and structural connectivity networks, and the hubs removal could lead to a worse performance of brain networks. |
| Other information | |  | | |
| Funding | 22 | Give the source of funding and the role of the funders for the present study and, if applicable, for the original study on which the present article is based | p18 | This work was supported by the National Natural Science Foundation of China [81401482, 81871337] and the Research Foundation of Artificial Intelligence Key Laboratory of Sichuan Province [2019RYJ04]. The funders had no role in study design, data collection and analysis, decision to publish, or preparation of the manuscript. |

*Give information separately for cases and controls in case-control studies and, if applicable, for exposed and unexposed groups in cohort and cross-sectional studies.

**Note:** An Explanation and Elaboration article discusses each checklist item and gives methodological background and published examples of transparent reporting. The STROBE checklist is best used in conjunction with this article (freely available on the Web sites of PLoS Medicine at http://www.plosmedicine.org/, Annals of Internal Medicine at http://www.annals.org/, and Epidemiology at http://www.epidem.com/). Information on the STROBE Initiative is available at www.strobe-statement.org.
