# Supplementary material for: Alterations of functional and structural connectivity in patients with brain metastases
Source: PLoS One. 2020 May 29;15(5):e0233833. doi: 10.1371/journal.pone.0233833 (PMC7259727; doi:10.1371/journal.pone.0233833)
Supplement: S1 Text — (PDF) [file pone.0233833.s002.pdf]

Text S1 Supplementary Methods

Brain network construction

Network node definition

To construct whole-brain functional and structural connectivity networks, we first define the nodes of brain network. We employed the automated anatomical labeling (AAL) template [1] to parcellate the whole brain into 90 cortical and subcortical regions (45 for each hemisphere, see S2 Table). To investigate the influence of tumor tissue on brain networks with different parcellated resolution, we further constructed a high-resolution network with 1024 regions, which is in line with previous studies [2-4]. The 1024 nodes were about the same size ($\approx$1.2 cm^3) and their borders aligned with those of the 90 AAL nodes. The two parcellation schemes were defined as AAL-90 and AAL-1024 respectively and applied to the following network analyses, as our previous study [4].

Edge definition of functional connectivity network

For rs-fMRI images of each subject, preprocessing was performed by using the Statistical Parametric Mapping software (SPM8, http://www.fil.ion.ucl.ac.uk/spm) and the toolbox for Data Processing & Analysis for Brain Imaging (DPABI V2.3, http://rfmri.org/dpabi) [5]. The first five volumes of the acquisition were discarded to ensure stabilization of scanner magnetization, and then the remaining 205 consecutive volumes were corrected for temporal differences in acquisition and realigned to the first volume for head motion correction. Next, individual T1-weighted structural images were co-registered to the mean functional images after realignment, and normalized to Montreal Neurological Institute (MNI) space. For the patients, we additionally used a cost-function modification to avoid transformation bias since the tumor tissue may lead to distortions during the T1 images normalization [6,7]. Briefly, we manually delineated each tumor on the T1-weighted structural images under the guidance of T2-weighted FLAIR images using MRIcron software (https://www.nitrc.org/projects/mricron). Then, we created a tumor mask with zeros in the tumor and ones outside the tumor, and used this tumor mask as an input-weighting mask during the step of T1 images normalization [8,9]. The transformation parameters from the T1 images normalization were further applied for the normalization of functional images to MNI space with a spatial resolution of 3×3×3 mm^3. After spatial normalization, the structural T1 images and functional images were visually inspected to ensure no potential image distortions existing due to the presence of tumor for each patient. Then, several nuisance parameters were regressed out of the data at each brain voxel, including the linear trend, Friston 24-parameter model, white matter signal, cerebrospinal fluid signal, global mean signal, and time points with frame displacement greater than 0.2 mm as well as two preceding and one subsequent frames [5,10,11]. The resulting residuals were temporally band-pass-filtered (0.01-0.1 Hz) to reduce effects of low-frequency drift and high-frequency noise [12,13], and used to substitute for the raw mean time series of the corresponding regions. By computing the Pearson correlation coefficients between the time series of every pair of regions, we acquired a temporal correlation matrix ($N \times N$, where N is 90 for AAL-90, and 1024 for AAL-1024) for each subject. In this study, we constructed weighted functional connectivity network to avoid loss of information. The absolute correlation coefficient $|r_{ij}|$ between region i and j was defined as the weighted edge w_{ij} of functional connectivity network. Since the mechanisms of negative functional connectivity are still less understood [14], we also constructed weighted functional connectivity network with only positive correlation coefficients and performed the following network analyses.

Edge definition of structural connectivity network

For DTI images, diffusion weighted images were first coregistered to the b0 image and corrected for eddy current distortions by using FMRIB's Diffusion Toolbox (FDT) 3.0 as implemented in FMRIB's Software Library (FSL) 5.0.9 (http://www.fmrib.ox.ac.uk/fsl). The diffusion tensor models were then estimated by using the Diffusion Toolkit [15], and fiber tracking was implemented in the DTI native space using Fiber Assignment by Continuous Tracking (FACT) algorithm [16]. Fiber tracking was terminated if it reached a voxel with fractional anisotropy less than 0.15 or turning angle between adjacent voxels greater than 35° [4,17]. For structural connectivity network, nodes were defined in the DTI native space [4,18]. To do this, T1-weighted anatomical images of each subject were first co-registered to the b0 image in the DTI native space using a linear transformation. The transformed T1-weighted images were then normalized to the T1-template of ICBM-152 MNI using a nonlinear transformation. For the patients, a cost-function modification was also additionally used to avoid tumor-induced transformation bias during the T1 images normalization [6,7]. Specifically, each tumor was manually delineated on the co-registered T1-weighted images under the guidance of T2-weighted FLAIR images using MRIcron software (https://www.nitrc.org/projects/mricron). A tumor mask with zeros in the tumor and ones outside the tumor was then created and used as an input-weighting mask during the normalization of co-registered T1-weighted images [8,9]. The derived transformation parameters were inverted and used to warp the AAL regions from MNI space to the DTI native space with nearest-neighbor interpolation, in line with previous studies [4,17,18]. In the DTI native space, region i and region j were thought to be connected when existing at least one fibre f with two end points located in both regions [18-20]. We constructed weighted structural connectivity network and defined its weighted edge $w(e)$ as: $w(e)=2/(S_i+S_j)\sum_{f\in F_e}1/l(f)$, which depicting the connection strength between region i and j [19-21]. S_i and S_j are two-dimension intersects of the individual's white matter with AAL region i and j, respectively [4,17]; $F(e)$ refers to the fibres set connecting regions i and j; $l(f)$ refers to the length of the fiber f. To normalize individual overall differences in connectivity strength, structural connectivity weights were further scaled by the maximum of this matrix for each subject [22].

Network topological analysis

We performed graph theoretical analysis to compute network topological properties via the Brain Connectivity Toolbox (http://www.brain-connectivtiy-toolbox.net) [23]. The global network properties such as connectivity strength S_{net}, normalized weighted clustering coefficient γ, normalized weighted characteristic path length λ and the small-worldness σ were evaluated. To determine the global role of each node in the brain networks, we also computed the nodal properties including nodal connectivity strength S_i, efficiency E_i and normalized betweenness centrality b_i. All formulas introduced below are based on $G=(N,K)$, a graph of network G with N nodes and K edges.

Global network properties

The connectivity strength is defined as $S_{net} = (1/N)\sum_{i \in G} S_i$ for weighted network. S_i is the nodal connectivity strength and computed as the sum of the edge weights w_{ij} linking to node i: $S_i = \sum_{j \in G} w_{ij}$. Thus, the connectivity strength of a network is the average of the nodal connectivity strength of all the nodes in the network.

The normalized clustering coefficient γ, normalized characteristic path length λ and the small-worldness σ are three important indices to evaluate whether a network has a small-world organization or not [24]. The normalized clustering coefficient is defined as $\gamma = C_{net}/C_{random}$, and the normalized characteristic path length is defined as $\lambda = L_{net}/L_{random}$, where C_{random} and L_{random} are the mean clustering coefficient and characteristic path length of 100 comparable random networks preserving the same number of nodes, edges and degree distribution as the real network [25]. For weighted network, the corresponding weights are redistributed. A small-world network has similar path length but higher clustering than a random network, that is $\gamma > 1$, $\lambda \approx 1$ [24]. The two conditions can also be summarized into a simple quantitative measurement, the small-worldness, $\sigma = \gamma/\lambda > 1$ [26,27].

The clustering coefficient C_{net} quantifies the extent of the local interconnectivity or cliquishness of information transfer on the network [24] and is defined as: $C_{net} = \frac{1}{N}\sum_{i \in G}\sum_{j,h \in G}\left(w_{ij}w_{ih}w_{jh}\right)^{1/3} / k_i\left(k_i - 1\right)$. w_{ij} is the weight between nodes i and j in the network, k_i is the degree of node i.

The characteristic path length L_{net} describes the extent of global efficiency or capability for parallel information propagation of the network. To overcome the problem of possibly disconnected network components, we computed L_{net} by a 'harmonic mean' length between pairs [28], that is, $L_{net} = \frac{1}{1/\left(N\left(N-1\right)\right)\sum_{i=1}^{N}\sum_{j \neq i}^{N} 1/L_{ij}}$. L_{ij} is the shortest path length between nodes i and j. The path length between nodes i and j is defined as the sum of the edge lengths along the path. For weighted network, each edge's length was obtained by computing the reciprocal of the edge weight, $1/w_{ij}$.

Nodal properties

The nodal connectivity strength S_i depicts the total level of connectivity of a node [23].

The nodal efficiency E_i characterizes the importance of the nodes for the communication within the network [29] and is defined as the inverse of the mean harmonic shortest path length between this node and the others in the network [30]: $E_i = \frac{1}{N-1}\sum_{i \neq j \in G}\frac{1}{L_{ij}}$.

The betweenness centrality B_i is the fraction of all shortest paths in the network that contain this node [31]. Here, we calculated the normalized betweenness centrality as: $b_i = B_i / [(N-1)(N-2)]$, which captures the influence of a node over information flow between other nodes in the network.

Accordingly, nodes with high connectivity strength, efficiency or betweenness centrality (> mean + SD) were considered as global hubs in the brain network [4,32].

Coupling analysis of functional-structural connectivity

We investigated the coupling between functional and structural connectivity networks for each subject. In line with our previous studies [4,17], we constrained the coupling analysis by the edges with present (non-zero) structural connectivity. Specifically, the non-zero structural connectivities were extracted to produce a vector of structural connectivity values. The values were then resampled into a Gaussian distribution [20,21]. Also, the corresponding functional connectivities were extracted to constitute a vector of functional connectivity values. The coupling of functional-structural connectivity was obtained by calculating Pearson's correlation of these two vectors.

Network analysis after tumors removal

To describe the influence of brain tumors on brain functional and structural networks, a simulated procedure was performed. Firstly, we identified the nodes infiltrated by brain tumors in each patient. Then, we computed the functional-structural connectivity coupling, and global network properties of functional and structural networks after removing these nodes and the corresponding connections for each patient.

Statistical analysis

The network topological parameters may change with the threshold selection. To comprehensively evaluate the tumor-induced network changes, we computed network topological properties using a range of cost thresholds (0.1≤cost≤0.26 for the AAL-90 scheme, 0.019≤cost≤0.036 for the AAL-1024 scheme). Here, the cost was computed as the ratio of the number of actual connections divided by the maximum possible number of connections in the network. The lower cost threshold was selected to ensure all brain networks were at least 80% connected and the mean degree of the network was larger than $2*\log(N)$ (N is 90 for the AAL-90 scheme and 1024 for the AAL-1024 scheme). The upper threshold was the maximum cost attained by structural brain networks. To avoid possible bias on network analysis from single threshold, we further computed the area under the curve (AUC) of network topological properties.

For group comparisons of global network properties and the coupling of functional-structural connectivity between controls and patients, two-sample two-tailed *t*-test was performed. The statistical significance for these group comparisons was determined using a nonparametric permutation test method [22]. The permutations were performed 5000 times to test whether the group differences were significant. A threshold of α=0.05 was used for testing network properties and coupling strength.

Furthermore, paired-samples *t*-test was employed to assess the difference of global network properties and the functional-structural connectivity coupling in patients before and after tumor removal. The

significance threshold strategy was carried out as above. To further evaluate the relationship of altered network properties and coupling strength with the tumor removal, Spearman correlation analysis was performed between these network alterations and the number of hubs infiltrated by tumors.

Reference

1. Tzourio-Mazoyer N, Landeau B, Papathanassiou D, Crivello F, Etard O, Delcroix N, et al. (2002) Automated anatomical labeling of activations in SPM using a macroscopic anatomical parcellation of the MNI MRI single-subject brain. Neuroimage 15: 273-289.
2. Fornito A, Zalesky A, Bullmore ET (2010) Network scaling effects in graph analytic studies of human resting-state FMRI data. Front Syst Neurosci 4: 22.
3. Zalesky A, Fornito A, Harding IH, Cocchi L, Yucel M, Pantelis C, et al. (2010) Whole-brain anatomical networks: does the choice of nodes matter? Neuroimage 50: 970-983.
4. Zhang Z, Liao W, Chen H, Mantini D, Ding JR, Xu Q, et al. (2011) Altered functional-structural coupling of large-scale brain networks in idiopathic generalized epilepsy. Brain 134: 2912-2928.
5. Yan CG, Wang XD, Zuo XN, Zang YF (2016) DPABI: Data Processing & Analysis for (Resting-State) Brain Imaging. Neuroinformatics 14: 339-351.
6. Brett M, Leff AP, Rorden C, Ashburner J (2001) Spatial normalization of brain images with focal lesions using cost function masking. Neuroimage 14: 486-500.
7. Andersen SM, Rapcsak SZ, Beeson PM (2010) Cost function masking during normalization of brains with focal lesions: still a necessity? Neuroimage 53: 78-84.
8. Brownsett SL, Warren JE, Geranmayeh F, Woodhead Z, Leech R, Wise RJ (2014) Cognitive control and its impact on recovery from aphasic stroke. Brain 137: 242-254.
9. Gooijers J, Beets IA, Albouy G, Beeckmans K, Michiels K, Sunaert S, et al. (2016) Movement preparation and execution: differential functional activation patterns after traumatic brain injury. Brain 139: 2469-2485.
10. Power JD, Barnes KA, Snyder AZ, Schlaggar BL, Petersen SE (2012) Spurious but systematic correlations in functional connectivity MRI networks arise from subject motion. Neuroimage 59: 2142-2154.
11. Power JD, Barnes KA, Snyder AZ, Schlaggar BL, Petersen SE (2013) Steps toward optimizing motion artifact removal in functional connectivity MRI; a reply to Carp. Neuroimage 76: 439-441.
12. Cordes D, Haughton VM, Arfanakis K, Carew JD, Turski PA, Moritz CH, et al. (2001) Frequencies contributing to functional connectivity in the cerebral cortex in "resting-state" data. AJNR Am J Neuroradiol 22: 1326-1333.
13. Foerster BU, Tomasi D, Caparelli EC (2005) Magnetic field shift due to mechanical vibration in functional magnetic resonance imaging. Magn Reson Med 54: 1261-1267.
14. Chen G, Xie C, Li SJ (2011) Negative functional connectivity and its dependence on the shortest path length of positive network in the resting-state human brain. Brain Connect 1: 195-206.

15. Wang R, Beener T, Sorensen AG, Weeden VJ (2007) Diffusion toolkit: a software package for diffusion imaging data processing and tractography. Proc Intl Soc Mag Reson Med 15: 3720.

16. Mori S, van Zijl PC (2002) Fiber tracking: principles and strategies - a technical review. NMR Biomed 15: 468-480.

17. Ding JR, An D, Liao W, Li J, Wu GR, Xu Q, et al. (2013) Altered functional and structural connectivity networks in psychogenic non-epileptic seizures. PLoS One 8: e63850.

18. Gong G, He Y, Concha L, Lebel C, Gross DW, Evans AC, et al. (2009) Mapping anatomical connectivity patterns of human cerebral cortex using in vivo diffusion tensor imaging tractography. Cereb Cortex 19: 524-536.

19. Hagmann P, Cammoun L, Gigandet X, Meuli R, Honey CJ, Wedeen VJ, et al. (2008) Mapping the structural core of human cerebral cortex. PLoS Biol 6: e159.

20. Hagmann P, Sporns O, Madan N, Cammoun L, Pienaar R, Wedeen VJ, et al. (2010) White matter maturation reshapes structural connectivity in the late developing human brain. Proc Natl Acad Sci U S A 107: 19067-19072.

21. Honey CJ, Sporns O, Cammoun L, Gigandet X, Thiran JP, Meuli R, et al. (2009) Predicting human resting-state functional connectivity from structural connectivity. Proc Natl Acad Sci U S A 106: 2035-2040.

22. van den Heuvel MP, Mandl RC, Stam CJ, Kahn RS, Hulshoff Pol HE (2010) Aberrant frontal and temporal complex network structure in schizophrenia: a graph theoretical analysis. J Neurosci 30: 15915-15926.

23. Rubinov M, Sporns O (2010) Complex network measures of brain connectivity: uses and interpretations. Neuroimage 52: 1059-1069.

24. Watts DJ, Strogatz SH (1998) Collective dynamics of 'small-world' networks. Nature 393: 440-442.

25. Maslov S, Sneppen K (2002) Specificity and stability in topology of protein networks. Science 296: 910-913.

26. Achard S, Salvador R, Whitcher B, Suckling J, Bullmore E (2006) A resilient, low-frequency, small-world human brain functional network with highly connected association cortical hubs. J Neurosci 26: 63-72.

27. Humphries MD, Gurney K, Prescott TJ (2006) The brainstem reticular formation is a small-world, not scale-free, network. Proc Biol Sci 273: 503-511.

28. Newman MEJ (2003) The structure and function of complex networks. SIAM Review 45: 167-256.

29. Bassett DS, Bullmore E (2006) Small-world brain networks. Neuroscientist 12: 512-523.

30. Achard S, Bullmore E (2007) Efficiency and cost of economical brain functional networks. PLoS Comput Biol 3: e17.

31. Freeman LC (1977) A set of measures of centrality based upon betweenness. Sociometry 40: 35-41.

32. He Y, Wang J, Wang L, Chen ZJ, Yan C, Yang H, et al. (2009) Uncovering intrinsic modular organization of spontaneous brain activity in humans. PLoS One 4: e5226.
