# Supplementary material for: Alterations of functional and structural connectivity in patients with brain metastases
Source: PLoS One. 2020 May 29;15(5):e0233833. doi: 10.1371/journal.pone.0233833 (PMC7259727; doi:10.1371/journal.pone.0233833)
Supplement: S1 Table — (PDF) [file pone.0233833.s003.pdf]

Table S1 Demographic and clinical characteristics of brain metastases

No.	Age/Sex	Metastasis number	Location	Size (mm^3)	Primary tumor
1	42/M	3	Right corpus callosum	1,739	Lung
			Left occipital	224	
			Right occipital	113	
2	47/M	1	Right parieto-occipital	8,040	Lung
3	63/M	1	Right occipital	66,977	Rectal
4	61/M	3	Left frontal	4,621	Lung
			Right frontal	596	
			Right primary motor	362	
5	67/F	1	Right frontal	3,026	Lung
6	68/M	1	Right primary motor	3,056	Lung
7	67/M	1	Right frontal	1,711	Lung
8	66/M	1	Left frontal	48	Lung
9	61/M	2	Right temporal	144	Lung
			Right cerebellum_8	3,282	
10	64/M	2	Left occipital	11,349	Lung
			Vermis_8	1,228	
11	60/M	3	Left parieto-occipital	1,211	Lung
			Left cerebellum_4_5	2,354	
			Left cerebellum_8	12,897	
12	54/M	1	Left occipital	155	Lung
13	60/M	1	Left fronto-parietal	464	Lung
14	61/M	1	Right Parietal	8,873	Lung
