# Supplementary material for: Alterations of functional and structural connectivity in patients with brain metastases
Source: PLoS One. 2020 May 29;15(5):e0233833. doi: 10.1371/journal.pone.0233833 (PMC7259727; doi:10.1371/journal.pone.0233833)
Supplement: S2 Table — (PDF) [file pone.0233833.s004.pdf]

Table S2 Regions of interest (ROI) in the AAL template

Region name	Abbreviation
Precentral	PreCG
Superior frontal gyrus (dorsolateral)	SFGdor
Superior frontal gyrus (orbital part)	ORBsup
Middle frontal gyrus	MFG
Middle frontal gyrus (orbital part)	ORBmid
Inferior frontal gyrus (opercular part)	IFGoperc
Inferior frontal gyrus (triangular part)	IFGtriang
Inferior frontal gyrus (orbital part)	ORBinf
Rolandic operculum	ROL
Supplementary motor area	SMA
Olfactroy cortex	OLF
Superior frontal gyrus (medial)	SFGmed
Superior frontal gyrus (medial orbital)	ORBsupmed
Rectus gyrus	REC
Insula	INS
Anterior cingulate gyri	ACG
Median cingulate gyri	MCG
Posterior cingulate gyrus	PCG
Hippocampus	HIP
Parahippocampal gyrus	PHG
Amygdala	AMYG
Calcarine fissure	CAL
Cuneus	CUN

Lingual gyrus	LING
Superior occipital gyrus	SOG
Middle occipital gyrus	MOG
Inferior occipital gyrus	IOG
Fusiform gyrus	FFG
Postcentral gyrus	PoCG
Superior parietal gyrus	SPG
Inferior parietal gyrus	IPG
Supramarginal gyrus	SMG
Angular gyrus	ANG
Precuneus	PCUN
Paracentral lobule	PCL
Caudate nucleus	CAU
Putamen	PUT
Pallidum	PAL
Thalamus	THA
Heschl gyrus	HES
Superior temporal gyrus	STG
Superior temporal gyrus, temporal pole	TPOsup
Middle temporal gyrus	MTG
Middle temporal gyrus, temporal pole	TPOmid
Inferior temporal gyrus	ITG

The abbreviations listed are those used in this paper, which differ slightly from the original abbreviations by Tzourio-Mazoyer (Tzourio-Mazoyer et al., 2002).
