# Supplementary figures and images for: Alterations of functional and structural connectivity in patients with brain metastases
Source: PLoS One. 2020 May 29;15(5):e0233833. doi: 10.1371/journal.pone.0233833 (PMC7259727; doi:10.1371/journal.pone.0233833)

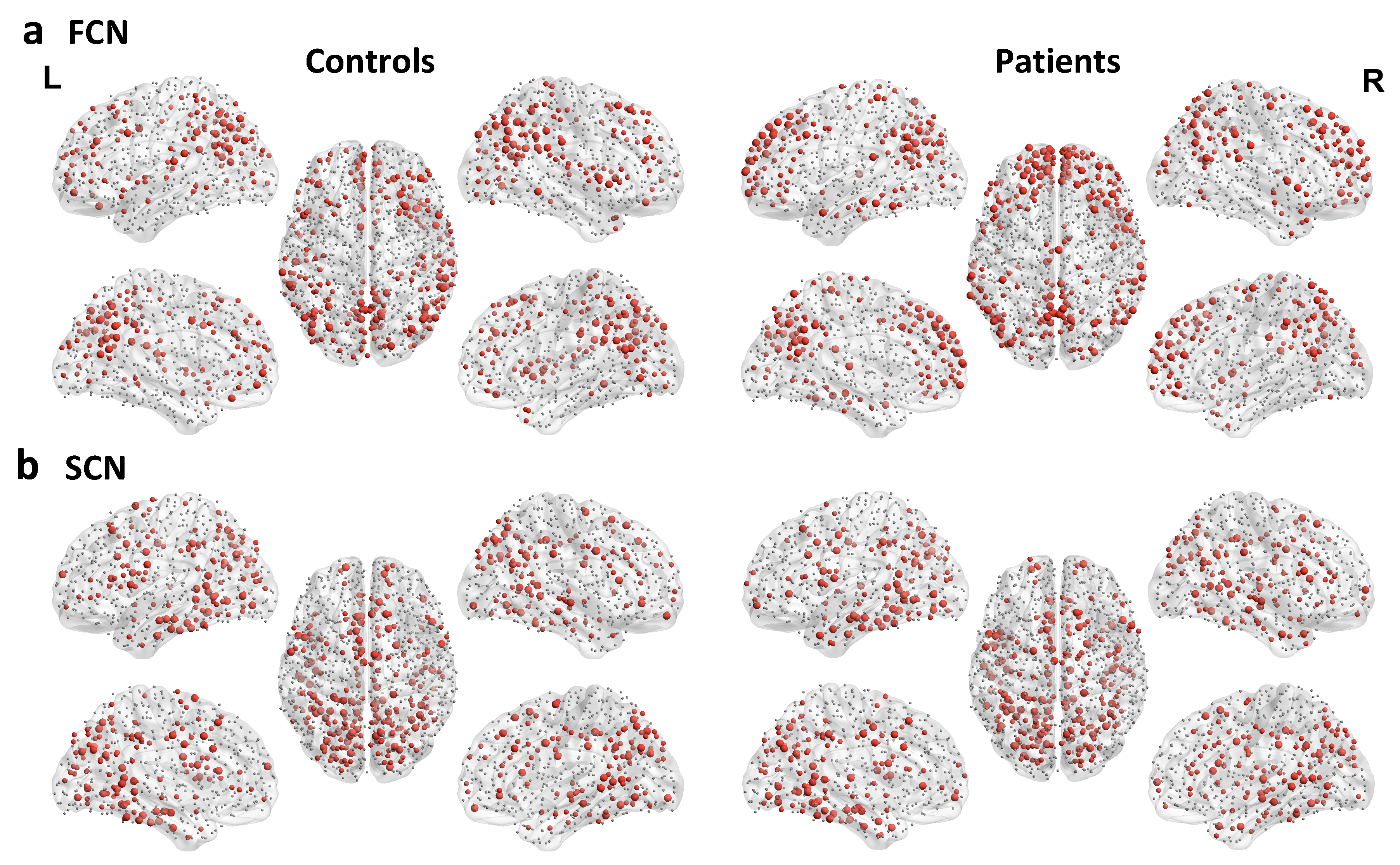

Supplement: S1 Fig — (a) functional connectivity network. (b) structural connectivity network. The results are visualized with the BrainNet viewer (NKLCNL, Beijing Normal University, China). The results of AAL-1024 scheme show similar pattern with those of AAL-90 scheme. The red spheres and the grey dots denote hub and non-hub regions, respectively. The nodal regions are located according to their centroid stereotaxic coordinates. FCN, functional connectivity network; SCN, structural connectivity network; L, left; R, right. (TIF) [file pone.0233833.s005.tif]

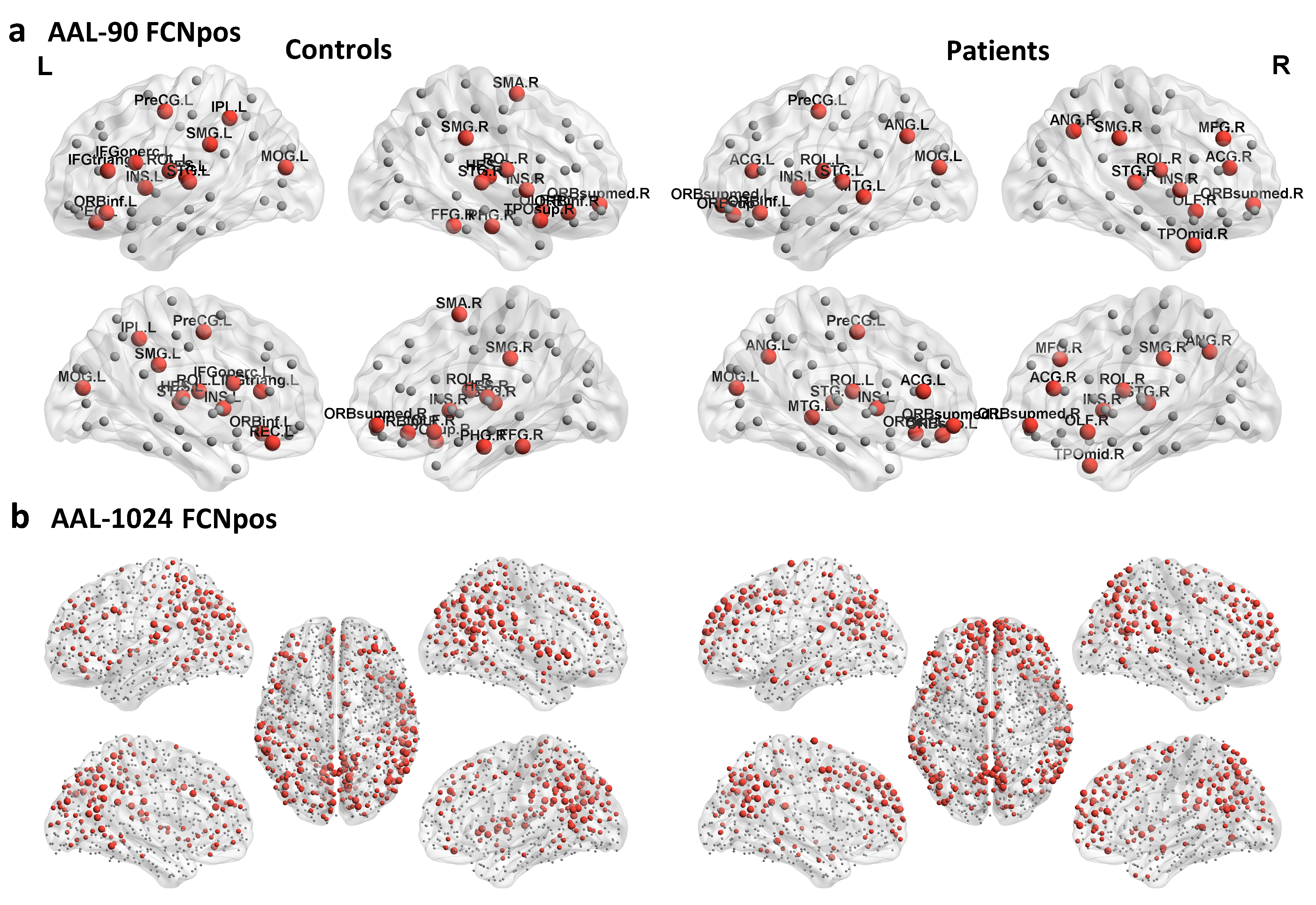

Supplement: S2 Fig — (a) under AAL-90 scheme. (b) under AAL-1024 scheme. The results are visualized with the BrainNet viewer (NKLCNL, Beijing Normal University, China). The hub distributions of AAL-1024 scheme is similar with those of AAL-90 scheme. The red spheres and the grey dots denote hub and non-hub regions, respectively. The nodal regions are located according to their centroid stereotaxic coordinates. FCNpos, functional connectivity network constructed by only positive correlation coefficients; L, left; R, right. (TIF) [file pone.0233833.s006.tif]

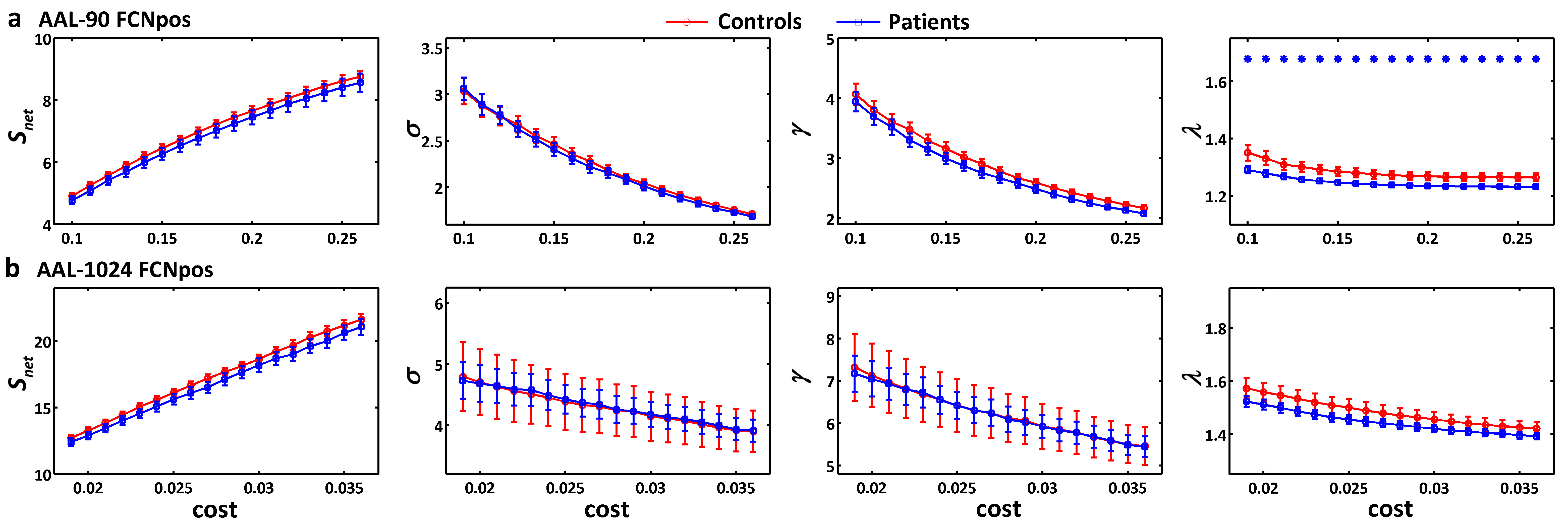

Supplement: S3 Fig — (a) under AAL-90 scheme. (b) under AAL-1024 scheme. The vertical bar indicates the standard deviation across subjects. The asterisks indicate the statistically significant difference between healthy controls and patients (permutation testing, p<0.05) FCNpos, functional connectivity network constructed by only positive correlation coefficients. (TIF) [file pone.0233833.s007.tif]

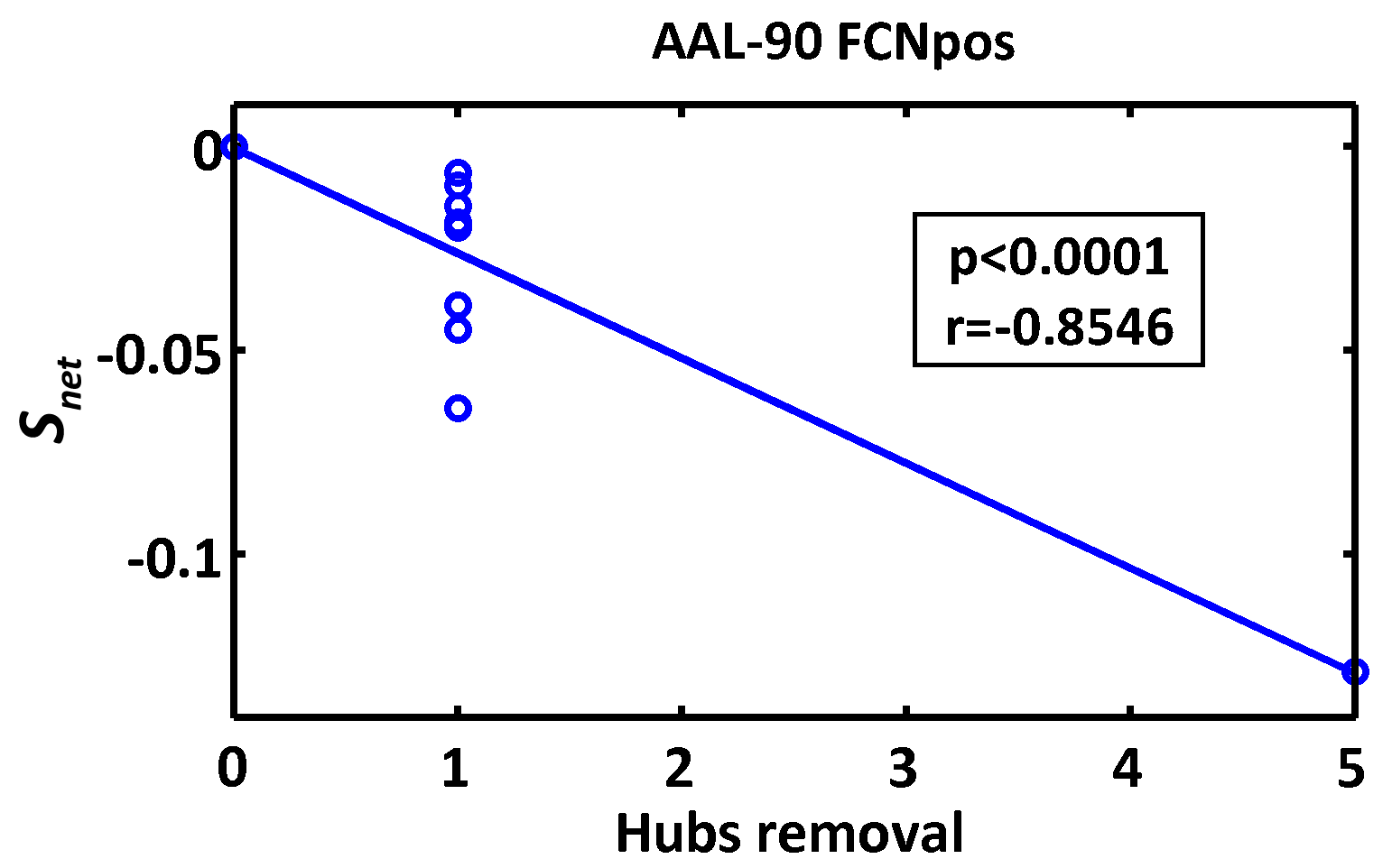

Supplement: S5 Fig — The altered network connectivity strength Snet of AAL-90 FCN constructed by only positive correlation coefficients negatively correlated with the removed hubs number (r = −0.8546, p<0.0001). FCNpos, functional connectivity network constructed by only positive correlation coefficients. (TIF) [file pone.0233833.s009.tif]
